# Neuroanatomical Relationships between Orexin/Hypocretin-Containing Neurons/Nerve Fibers and Nicotine-Induced c-Fos-Activated Cells of the Reward-Addiction Neurocircuitry

**DOI:** 10.4172/2329-6488.1000273

**Published:** 2017-07-20

**Authors:** Ozra Dehkordi, Jed E Rose, Martha I Dávila-García, Richard M Millis, Samar Ali Mirzaei, Kebreten F Manaye, Annapurni Jayam-Trouth

**Affiliations:** 1 Department of Neurology, Howard University Hospital, Washington, DC, USA; 2 Department of Physiology and Biophysics, Howard University College of Medicine, Washington, DC, USA; 3 Department of Psychiatry, Duke University Medical Center, Durham, NC 27705, USA; 4 Department of Pharmacology, Howard University College of Medicine, Washington, DC, USA; 5 Department of Medical Physiology, College of Medicine, American University of Antigua, Antigua and Barbuda, West Indies

**Keywords:** Addiction, Dopamine, Nicotine, Orexin, Reward, Ventral Tegmental Area (VTA)

## Abstract

Orexin/hypocretin-containing neurons in lateral hypothalamus (LH) are implicated in the neurobiology of nicotine addiction. However, the neuroanatomical relationships between orexin-neurons/nerve fibers and nicotine-activated cells within the reward-addiction neurocircuitry is not known. In the present study in mice, we first used c-Fos immunohistochemistry to identify CNS cells stimulated by an acute single injection of nicotine (NIC, 2 mg/kg, IP). Sequential double-labelling was then performed to identify the location of orexin-containing neurons and nerve fibers with respect to NIC-induced c-Fos activated cells and/or tyrosine hydroxylase (TH) immunoreactive (IR) cells of the mesocorticolimbic reward-addiction pathways. Orexin-IR nerve fibers and terminals were detected at multiple sites of the NIC reward-addiction circuitry in close apposition to, and intermingled with, NIC-induced c-Fos-IR cells of locus coeruleus (LC), ventral tegmental area (VTA), nucleus accumbens (Acb), LH and paraventricular thalamic nucleus (PVT). Double-labelling of orexin with TH showed frequent contact between orexin-IR nerve fibers and noradrenergic cells of LC. However, there was infrequent contact between the orexinergic fibers and the TH-expressing dopaminergic cells of VTA, dorsal raphe nucleus (DR), posterior hypothalamus (DA11), arcuate hypothalamic nucleus (DA12) and periventricular areas (DA14). The close anatomical contact between orexinergic nerve fibers and NIC-activated cells at multiple sites of the reward-addiction pathways suggests that orexinergic projections from LH are likely to be involved in modulating activity of the neurons that are directly impacted by acute administration of nicotine.

## Introduction

Orexins/hypocretins are excitatory neuropeptides synthesized by neurons located exclusively in three hypothalamic regions: Lateral Hypothalamus (LH), perifornical area and dorsomedial hypothalamus [1,2]. Although these neurons represent a relatively small number of cells, they project extensively to various brain regions and are implicated in a number of physiological functions, such as arousal, cognition, stress, appetite, metabolism and addiction [1,3-9]. The idea that orexin-containing neurons may be implicated in the neurocircuitry of addiction initially emerged from studies documenting the involvement of orexin in natural rewards such as food and sex [10,11] and orexinergic projections to structures of the reward-addiction pathways [12-17]. Numerous studies have shown that orexin plays a critical role in drug addiction and reward-related behaviours of several addictive drugs, including nicotine [6-9,16,18]. Orexin appears to be involved in the reinforcing properties of nicotine, reinstatement of nicotine-seeking behaviours and anxiogenic effects of acute nicotine [7,19] Immunohistochemical studies have shown that orexinergic neurons are activated by both acute nicotine exposure and nicotine withdrawal [6,20]. We have recently demonstrated that acute single intraperitoneal (IP) injection of nicotine and its peripherally acting analogue, nicotine pyrrolidine methiodide, stimulate neurons at multiple regions of the CNS including LH [21,22]. However, the neuroanatomical relationships between nicotine-activated cells and orexinergic nerve fibers at LH and other brain regions is not known. Thus, one of the objectives of the present study was to determine if NIC-activated areas of the reward-addiction pathways are the targets of orexinergic innervation. Since noradrenergic cells of locus coeruleus (LC) and dopaminergic cells of ventral tegmental area (VTA) and other brain regions are among the main neurochemical substrates known to be impacted by nicotine, we also evaluated noradrenergic and dopaminergic cells of the reward-addiction pathways as potential targets of orexinergic innervation in the CNS.

## Material and Methods

### Subjects

Experiments were performed in adult CD-1 mice weighing 20-25 g. All procedures including the anesthesia and surgery were reviewed and approved by the Institutional Animal Care and Use Committee (IACUC) of Howard University. All efforts were made to minimize the number of animals used and their suffering.

### Experimental protocol

Animals (N=20) were housed at room temperature (22-24°C) with water and food freely available. To reduce the nonspecific effects of handling and experimental environment, animals were handled daily and exposed to the same conditions as during the actual experiments. Following an adaptation period of 3-4 days, the mice received IP injection of physiological saline (control; PS) and/or nicotine hydrogen tartrate salt (NIC: 2 mg/kg; Sigma Aldrich, St. Louis, MO). NIC was dissolved in PS (vehicle) and injected in volumes of 0.2 ml/injection. Two hours after IP injection of PS and/or NIC, the mice were anesthetized with 5% isoflurane and perfused transcardially with saline, followed by 4% paraformaldehyde in 0.1 M Phosphate Buffer (PB) at pH 7.4. After perfusion, the brains were post fixed in 4% paraformaldehyde for 1 h and then cryoprotected in a 30% sucrose solution for a minimum of 2 d. Transverse sections of the brain were cut at 40 μm using a Bright OTF Cryostat (Hacker Instruments and Industries) and stored in 0.5% sodium azide in 0.1 M PB (pH 7.4).

### Immunohistochemistry

Immunohistochemical procedures were performed using free floating sections as follows: Briefly, 1-in-5 series of brain sections extending from bregma –5.63 mm to bregma 2.33 mm [23] were rinsed 3 times in 0.1 M phosphate-buffered saline (PBS) at pH 7.4. Nonspecific binding was blocked by incubating the tissues overnight in loading buffer containing 2% normal donkey serum (NDS, Santa Cruz Biotechnology, Inc., Santa Cruz, CA) and 0.3% Triton X-100. Tissues were then washed and processed for sequential double labelling of orexin immunoreactive (IR) neurons and nerve fibers with either NIC-induced c-Fos, PS-induced c-Fos, or TH-IR cells according to the following protocols.

### Double labelling of orexin and c-Fos

For double labelling of c-Fos and orexin, tissues were washed and incubated with a PBS cocktail consisting of 2% NDS, 0.3% Triton X-100, rabbit anti-c-Fos (1:100 of Cat#Sc-52, Santa Cruz Biotechnology, Inc. and/or 1:1000 of Cat#ABE457 Millipore Corporation, Temecula, CA) and goat anti-orexin (1:100 of Cat#Sc-8070 Santa Cruz Biotechnology, Inc.) antibodies at 4°C for 48-72 h. The sections were then washed and incubated in Alexa Fluor 594 donkey anti-rabbit secondary antibody (1:100; Jackson ImmunoResearch Laboratories Inc.) in 0.1 M PBS for 2½ h. After washing in PBS, sections were incubated with Cy™ 5 donkey anti-goat secondary antibody (1:100; Jackson ImmunoResearch Laboratories Inc.) for 2½ h. Finally, the sections were rinsed in PBS and cover-slipped using Vecta Shield (Vector Laboratories) anti-fade mounting media.

### Double labelling of orexin and tyrosine hydroxylase

For double labelling of orexin and tyrosine hydroxylase (TH), tissues were washed and incubated with a PBS cocktail consisting of 2% NDS, 0.3% Triton X-100, goat anti-orexin (1:100 of Cat #Sc-8070 Santa Cruz Biotechnology Inc.) and mouse anti-TH (TH: 1:1000; Cat #T1299, Sigma-Aldrich) antibodies at 4°C for 48 h. The sections were then washed and incubated in Alexa Fluor 594 donkey anti-goat secondary antibody (1:100; Jackson ImmunoResearch Laboratories Inc.) in 0.1M PBS for 2½ h. After washing in PBS, sections were incubated with Alexa-Fluor 488 donkey anti-mouse secondary antibody (1:100, Jackson) for 2½ h. Finally, the sections were rinsed in PBS and cover-slipped using Vecta Shield (Vector Laboratories) anti-fade mounting media.

Controls for each experiment were performed to determine whether the primary or the secondary antibodies produced false-positive results. The controls involved omission of the primary and/or secondary antisera to eliminate the corresponding specific labelling. Nonspecific activation of c-Fos was assessed by evaluating the CNS expression of c-Fos in animals receiving IP injection of PS.

### Data analysis

High resolution fluorescent images were acquired using Nikon (Nikon Instruments, Melville, NY) microscope equipped with the adequate filter systems to observe the red, green and CY5 fluorescence. Co-localization of orexin IR cells with c-Fos IR cells, TH IR adrenergic, and/or dopaminergic cells was detected by sequential capturing of the images, alternating between filters appropriate for each labelling and by analyzing the merge images of the exact same sites. Images from all the brain regions of interest were captured at 4×, 10× and 20× magnification and minor adjustments of brightness and contrast were made using Adobe Photoshop CS3.

### Cell counting

A semi-quantitative estimate of the total number of NIC-, or PS-induced c-Fos-activated cells in LH that were orexin-immunoreactive was performed as follows: Two 40 μm sections from different rostro-caudal levels of LH corresponding to bregma -1.55 and -2.03 mm (Paxinos and Franklin, 2013) were selected for each animal in each group (N=4). We counted the total numbers of NIC-, or PS-induced c-Fos IR cells, the total number of cells that exhibited orexin immunoreactivity and the number of c-Fos IR cells in LH that co-expressed orexin. We then calculated the percentage of orexin IR cells that expressed c-Fos. The data were then analyzed using one-way ANOVA followed by a post-hoc t-test. The results were considered significant at P<0.05.

## Results

### Interaction between orexin-containing nerve fibers and NIC-induced c-Fos immunoreactive cells

**Figure 1:**
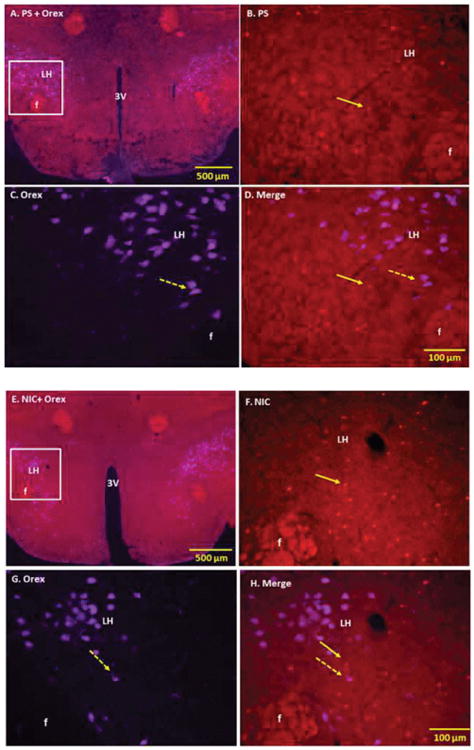
Double immunofluorescence staining demonstrating the location of nicotine (NIC) and physiological saline (PS) induced c-Fos immunoreactive (IR) neurons with respect to orexin (Orex) IR cells of lateral hypothalamus (LH). Panel A Low power fluorescent image showing PS-induced c-Fos and Orex IR cells of LH Panels B-D: High power images showing PS-induced c-Fos IR cells (B), Orex IR cells (C) and merged images of c-Fos with Orex in LH (D). Panel E: Low power fluorescent image showing NIC-induced c-Fos and Orex IR cells of LH Panels F-H: High power images showing NIC-induced c-Fos IR cells (F), Orex IR cells (G) and merged images of c-Fos with Orex in LH (H). The number of Orex IR cells activated by NIC was small and did not reach statistical significance Solid arrows point to representative c-Fos IR and broken arrows to Orex IR neurons Squares indicate magnified areas F: Fornix; 3V 3^rd^ Ventricle.

Consistent with previous immunohistochemical studies, orexin-containing neurons were present in the hypothalamus (HP) and they were restricted to the perifornical region, dorsomedial hypothalamic nucleus (DMH) and dorsal and lateral hypothalamus (DH, LH). Acute IP injection of NIC produced c-Fos activation of various intensities in different regions of HP, including areas overlapping the orexinergic cells of LH (Figure 1).

Double labelling of orexin-IR cells with NIC-induced c-Fos activated cells showed that a small fraction of orexin-containing neurons were activated by an acute single injection of NIC (15.1% ± 9.7 SD); however, the number of orexin-IR cells that were activated by NIC was not statistically different than the number of orexin-IR cells activated by the saline vehicle (9.3% ± 5.9 SD).

**Figure 2:**
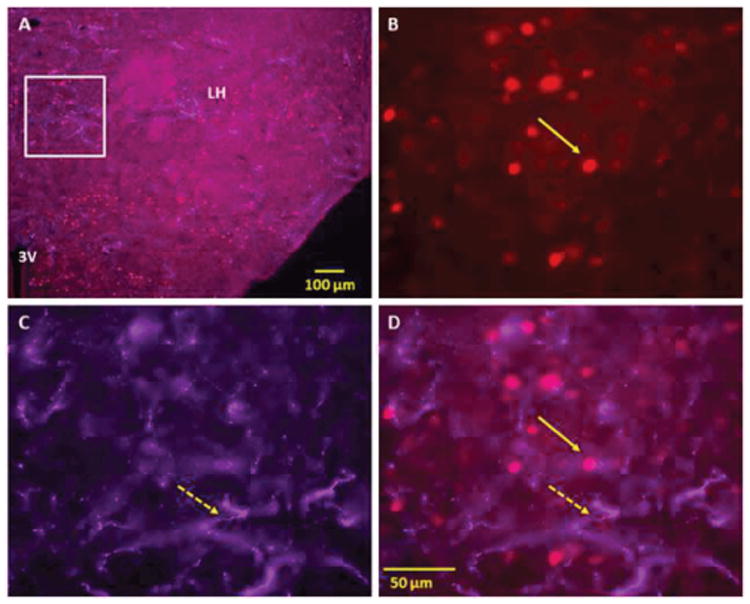
Double immunofluorescence staining demonstrating the location of orexin (Orex) immunoreactive (IR) nerve fibers with respect to nicotine (NIC) induced c-Fos IR cells of lateral hypothalamus (LH). Panel A: Low power fluorescent image showing NIC-induced c-Fos IR and Orex IR nerve fibers in LH Panels B-D: High power images showing NIC-induced c-Fos IR cells (B), Orex IR nerve fibers (C) and merged images of c-Fos with Orex IR fibers (D). Orex IR nerve fibers are seen in areas overlapping NIC induced c-Fos activated cells of LH and several other hypothalamic regions. Solid arrows point to representative c-Fos IR and broken arrows to orexin IR nerve fibers. Square indicates magnified area (3V 3^rd^ Ventricle).

Orexin-containing nerve fibers were seen intermingled with NIC–induced c-Fos activated cells at LH and at multiple other sites in hypothalamus, including the posterior hypothalamic area, ventromedial hypothalamic nucleus, arcuate hypothalamic nucleus and paraventricular hypothalamic nucleus (Figure 2).

**Figure 3:**
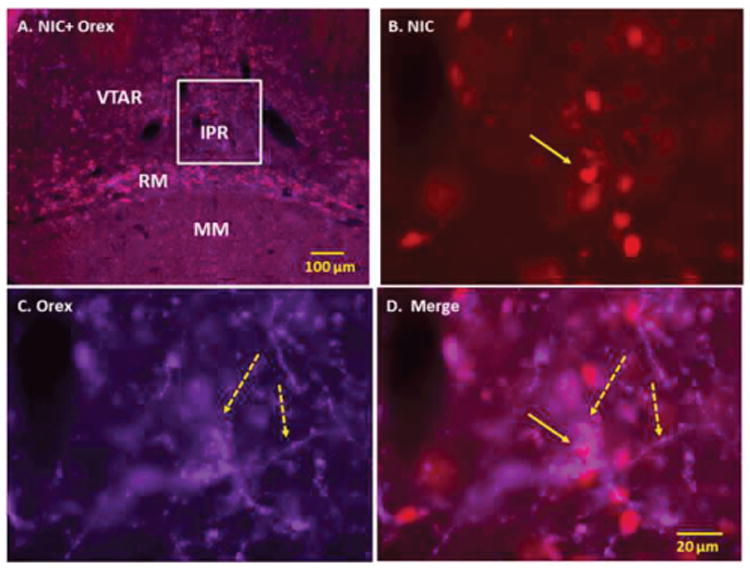
Double immunofluorescence staining demonstrating the location of orexin (Orex) immunoreactive (IR) nerve fibers with respect to nicotine (NIC) induced c-Fos IR cells of ventral tegmental area (VTA). Panel A: Low power fluorescent image showing NIC-induced c-Fos IR and Orex IR nerve fibers in VTA. Panels B-D: High power images showing NIC-induced c-Fos IR cells (B), Orex IR nerve fibers (C) and merged images of c-Fos with Orex IR fibers (D). Orex IR nerve fibers are seen in close contact with NIC activated cells of VTA. Solid arrows point to representative c-Fos IR and broken arrows to orexin IR nerve fibers. Square indicates magnified area. Abbreviations: RM: Retromamillary Nucleus; IPR: Interpeduncular Nucleus Rostral; MM: Medial Mammillary Nucleus; VTAR: Ventral Tegmental Area Rostral.

Orexin-containing nerve fibers were present at several other sites, including additional areas that were activated by acute injection of NIC. These sites include VTA, dorsal raphe nucleus (DR), periaqueductal gray (PAG), LC, paraventricular thalamic nucleus (PVT), nucleus accumbens (Acb) and lateral septal nucleus (LS). In VTA, substantial overlapping was observed between high-density orexin-containing nerve fibers and NIC-induced c-Fos IR cells in areas overlaying retromamillary nucleus (RM), ventral tegmental area rostral (VTAR) and interpeduncular nucleus rostral (IPR). Varicose orexin–containing nerve fibers at these sites were frequently seen in contact with, and in close apposition to, c-Fos IR NIC-activated cells (Figure 3).

In caudal VTA, orexin-containing nerve fibers were detected at areas overlapping interpeduncular nucleus rostral (IPR), paranigral nucleus (PN), parainterfascicular nucleus (PIF) and interfascicular nucleus (IF). The scarcely distributed c-Fos IR cells found in these areas did not appear to make contact with the orexin-IR nerve fibers (data not shown).

**Figure 4:**
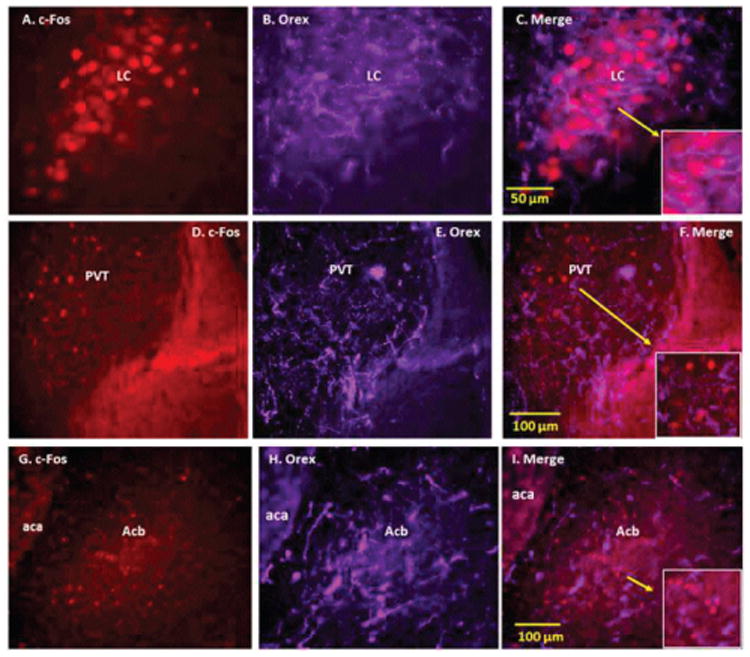
Double immunofluorescence staining demonstrating the location of orexin (Orex) immunoreactive (IR) nerve fibers with respect to nicotine (NIC) induced c-Fos IR cells of locus coeruleus (LC), paraventricular thalamic nucleus (PVT) and nucleus accumbens (Acb). Panels A-C: NIC-induced c-Fos IR cells (A), Orex IR fibers (B) and merged images of c-Fos with Orex nerve fibers (C) in LC. Panels D-F: NIC-induced c-Fos IR cells (D), Orex IR fibers (E) and merged images of c-Fos with Orex IR nerve fibers (F) in PVT. Panels G-I: NIC induced c-Fos IR cells (G), Orex IR fibers (H) and merged images of c-Fos with Orex IR nerve fibers (I) in Acb Orex IR nerve fibers are seen to intermingle with c-Fos induced NIC activated cells of LC, PVT and Acb and in certain areas such as LC there is close anatomical contact between Orex IR nerves and NIC activated cells Arrows point to magnified areas Abbreviations: aca: Anterior Commissure, Anterior Part.

In LC, orexin-IR nerve fibers formed a very dense network in close apposition to the c-Fos activated cells (Figure 4; Panels A-C). A dense network of orexin-IR fibers was also detected among the NIC-induced c-Fos-activated cells in PVT (Figure 4; Panels D-F). In this area, some varicose orexin-IR fibers were observed in close apposition to c-Fos-activated cells. Orexin-IR fibers were also observed in areas overlapping the NIC-induced c-Fos-activated sites of Acb (Figure 4; Panels G-I), DR, PAG and LS (data not shown).

### Interaction between orexin-containing nerve fibers and noradrenergic and dopaminergic cells

**Figure 5:**
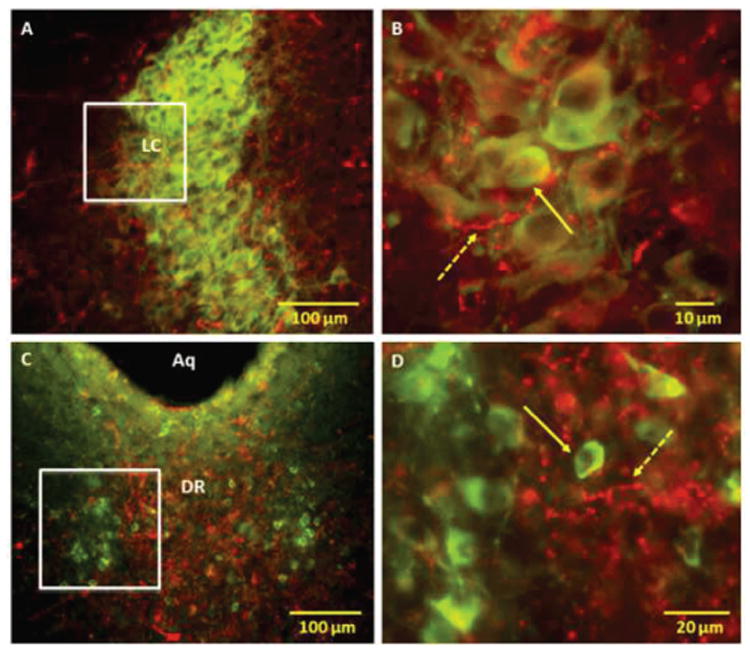
Fluorescent microscopy images of representative brainstem sections demonstrating the location of orexin (Orex) immunoreactive (IR) nerve fibers with respect to tyrosine hydroxylase (TH) IR cells of locus coeruleus (LC) and dorsal raphe (DR). Panels A and B: Low and high power merged images showing the location TH IR with respect to Orex IR nerve fibers in LC; Orex IR nerve fibers were seen in close contact and intermingled with TH IR noradrenergic cells of LC. Panels C and D: Low and high power merged images showing the location TH IR dopaminergic cells with respect to Orex IR nerve fibers in DR; scarcity of anatomical contact between Orex IR nerve fibers and TH IR cells implies that dopaminergic cells may not be the main target of orexin at this site Solid arrows point to representative c-Fos IR and broken arrows to orexin IR nerve fibers Squares indicate magnified areas.

An extensive network of orexin-IR nerve fibers was also detected in close apposition to noradrenergic TH-IR cells in LC (Figure 5; Panels A and B). In DR, orexin-IR fibers were seen in areas overlapping the TH-IR dopaminergic cells. However, only a small number of these fibers were found contacting TH-IR cells (Figure 5; Panels C and D).

**Figure 6:**
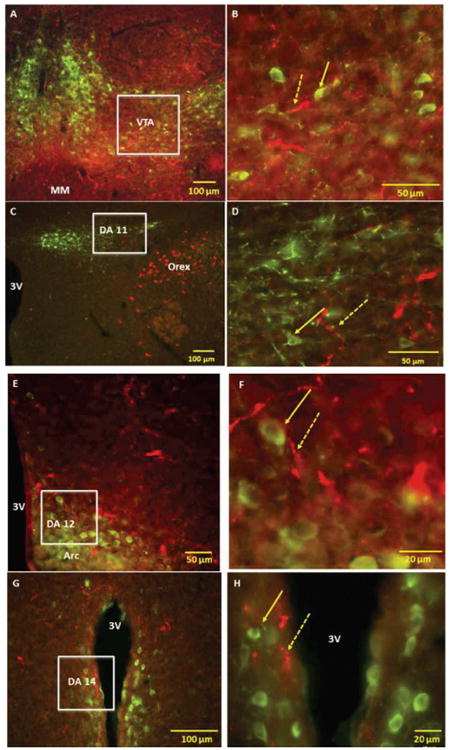
Fluorescent microscopy images of representative brain sections demonstrating the location of orexin (Orex) immunoreactive (IR) nerve fibers with respect to tyrosine hydroxylase (TH) IR dopaminergic cells of ventral tegmental area (VTA), DA11 cells of posterior hypothalamus, DA12 cells of arcuate hypothalamic nucleus (Arc) and DA14 of periventricular areas Panels A and B: Low and high power merged images showing the location TH IR cells in VTA with respect to Orex IR nerve fibers; Orex IR nerve fibers are present but make infrequent contact with dopaminergic cells of VTA Panels C and D: Low and high power merged images showing the location of TH IR cells of posterior hypothalamus (DA11) with respect to Orex IR neurons and nerve fibers; Orex IR cells are seen in the vicinity of DA11 cells; small number of Orex IR nerve fibers are seen in areas overlapping the DA11 cells Panels E and F: Low and high power merged images showing the location TH IR cells of periventricular areas (DA14) with respect to Orex IR nerve fibers; small number of Orex IR nerve fibers were detected in areas overlapping DA14 cells Solid arrows point to representative c-Fos IR and broken arrows to orexin IR nerve fibers Squares indicate magnified areas Abbreviations: 3V 3^rd^ Ventricle.

In VTA, orexin-IR fibers were seen in areas overlying the TH-IR dopaminergic cells (Figure 6; Panels A and B). However, the majority of these fibers did not appear to make anatomical contact with the TH-IR cells and are therefore likely to be en passant “fibers of passage.” In hypothalamus, DA11 dopaminergic cells were seen medial and dorsal to orexin-IR cells and very few orexin–IR nerve fibers were detected among the DA11 cells (Figure 6; Panels C and D). Orexin-IR fibers were also detected among the DA12 (Figure 6; Panels E and F) and DA14 dopaminergic cells (Figure 6; Panels G and H); however, they did not appear to make contact with these cells.

## Discussion

The main finding of the present study is that orexinergic neurons of LH innervate the structures that are also activated by acute administration of NIC. The apparent anatomical contacts between orexin-IR and NIC-induced c-Fos IR cells in LC, VTA, PVT and other structures of the reward-addiction circuitry implies that cells that are directly impacted by acute NIC may be the targets of orexin in the CNS.

Multiple other studies suggest that orexinergic cells of LH may play an important role in drug addiction and the reward-related behaviours of NIC and other psychostimulant drugs [7-9,16,18]. For example, blockade of orexinergic transmission at hypocretin-1 (Hcrt-1; orexin-1) receptors decreases NIC self-administration in rats, with no effects on food responding [7,24]. Blockade of this receptor also abolishes the stimulatory effects of NIC on brain reward circuitries [7]. Local administration of the orexin-1 receptor antagonist SB-334867 to insular cortex, a CNS structure implicated in smoking behaviours in humans, also decreases NIC self-administration [7]. In addition to its role in the reinforcement properties of NIC, intracerebroventricular infusion of orexin is reported to cause reinstatement of previously extinguished NIC-seeking behaviour in mice [19]. Others have reported that orexin-2 receptor antagonists may attenuate the relapse related to environmental stimuli previously associated with self-administration of NIC [25]. Studies in human smokers have reported that orexin plasma levels were inversely correlated to NIC craving [26]. Our neuroanatomical data corroborate these studies concerning the importance of orexin in drug addiction and, more specifically, suggests that orexinergic projections from the lateral hypothalamus to NIC activated regions may modulate the pharmacological effects of nicotine on the CNS.

Previous studies have reported that a small percentage of orexinergic cells in LH are activated by acute NIC in rats [6]. These investigators have also reported that glutamatergic and cholinergic inputs to LH may mediate NIC activation of orexin neurons in this region [27]. In the present study in mice, the number of orexinergic cells activated by acute NIC was small, and did not reach statistical significance. Since the effect of NIC on orexin neurons is shown to be dose-dependent in rats [6], it is plausible that the NIC doses we used in mice, with different metabolisms than rats, may not have been sufficient to activate more orexin cells in LH. Chronic NIC is known to upregulate expression of orexin and its receptors [28]. Thus, if NIC activation of orexin neurons is via glutamatergic and cholinergic mechanisms, as reported by Pasumarthi and Fadel [27], a single acute injection of NIC may not have provided sufficient trans-synaptic input to activate the orexin cells. It is noteworthy that even in the previously reported study [6], the vast majority of orexinergic cells did not respond to acute administration of NIC.

### Orexinergic innervation of dopaminergic and non-dopaminergic NIC-activated cells in VTA

VTA, an important brain site that regulates the rewarding actions of abused drugs, is also known to receive orexinergic innervation. Retrograde labelling shows that 20% of VTA projecting cells from LH express orexin-A-like immunoreactivity [14]. Immunohistochemical studies demonstrate the presence of orexin-1 and orexin-2 receptors in dopaminergic and non-dopaminergic cells of VTA [13,15,16]. Electrophysiological studies indicate that orexin can activate both dopaminergic and non-dopaminergic cells of VTA by direct postsynaptic mechanisms [15]. Orexin administration to VTA increases dopamine efflux in Acb and medial prefrontal cortex [16,29]. Consistent with these findings, our anatomical data demonstrate the presence of orexinergic projections throughout the rostral-caudal extension of VTA. In rostral VTA, we found these fibers in close proximity, and apposition to, NIC-induced c-Fos activated cells. However, the paucity of these fibers in the vicinity of TH-IR cells implies that dopaminergic cells may not be the primary targets of orexinergic innervation in VTA. Our findings corroborate previous immunohistochemical studies in rats reporting infrequent input of orexinergic nerve fibers to dopaminergic neurons of VTA and absence of synaptic contacts between the dopaminergic cells and orexin-IR nerve fibers [30]. The close anatomical contact between orexinergic nerve fibers and non-dopaminergic NIC-induced c-Fos activated cells in VTA implies that cells activated by NIC may be one of the targets of orexinergic innervation at these sites. Our anatomical data do not fully support electrophysiological experiments reporting that orexin activates both dopaminergic and non-dopaminergic cells of VTA via a direct postsynaptic mechanism [15]. The presence of orexin-1 and orexin-2 receptors on dopaminergic cells and the apparent absence of anatomical synapses between orexinergic nerve fibers and dopaminergic cells in VTA suggest that orexinergic innervation of dopaminergic cells may occur via functional, and not anatomical, synapses. Another possibility is that orexinergic innervation of non-dopaminergic cells, including NIC-induced c-Fos activated cells, may lead to trans-synaptic activation of dopaminergic cells in VTA.

The infrequent anatomical contact between orexinergic fibers and dopaminergic cells in other regions of the CNS, such as those in DR, DA11 cells of posterior hypothalamus, DA12 cells of arcuate hypothalamic nucleus (Arc) and DA14 of periventricular areas, implies that dopaminergic cells may not be the targets of orexin innervation at these sites. Among these regions, DR exhibits the most intense orexinergic innervation. DR is a heterogeneous structure that contains multiple other cell types including serotonergic, GABAergic and glutamatergic neurons [31,32]. Thus, intense orexinergic innervation of this site, as demonstrated in the present study, implies that other cell types are the potential targets of orexin at DR. Indeed, previous studies have demonstrated that serotonergic neurons, one of the main neural substrates of arousal, are the targets of orexinergic innervation in DR [33].

### Orexinergic innervation of other NIC-activated regions of the reward-addiction circuitry

LC and PVT are other sites within the reward-addiction circuitry that we identified as having intense orexinergic innervation and NIC-induced c-Fos activation. The proximity of orexinergic nerve fibers to NIC-induced c-Fos-IR cells in LC, the majority of which are noradrenergic, suggests that orexinergic innervation also modulates activity of LC neurons. These data are supported by previous anatomical studies in orexin-1 green fluorescent protein transgenic reporter mice, demonstrating extensive co-expression of orexin-1 receptors with the TH-IR cells in LC [34]. Functional studies show that exogenous application of orexin-A increases the firing rate of LC neurons and, when administered at the onset of sleep, induces arousal [35]. PVT, a neuronal structure that plays an important role in arousal, attention and awareness [36] is also a site that mediates the effects of NIC on arousal and attention [17]. Thus, orexinergic projections to NIC-activated cells of PVT and LC, as demonstrated in the present studies, may enhance arousal and attention responses to NIC.

Other reward related regions that are activated by NIC and receive orexinergic innervations are LS and Acb. LS, a key component of the limbic system, is involved in the regulation of a wide variety of functions including learning and memory, stress, anxiety and reward [37-42]. How orexin innervation of LS may affect these functions is not known. In the present study, despite intense orexinergic innervation of LS, we detected a small number of these fibers in the vicinity of NIC-activated cells. This finding suggests that these cells may not be the primary targets of orexin at this site.

Acb has received distinct attention because virtually all addictive drugs, including NIC, induce dopamine release at this site [43-45]. Acb also participates in control of behaviors related to natural reinforcers, such as feeding and sexual behaviors [46-50]. However, the mechanism by which orexinergic innervation of NIC-activated regions of Acb affects NIC- related addictive behavior is not known. Previous studies have shown that orexinergic innervation of Acb could promote feeding, drinking [51] and drug intake [52]. Lei et al. [52] have demonstrated that activation of orexin-1 receptors within Acb promotes alcohol intake in mice. Others have reported that orexin-A in Acb is involved in feeding and drinking, but not alcohol preference [51]. In addition, blockade of orexin receptor-1 in Acb decreases the expression of morphine-induced conditioned place preference (CPP) and shortens the extinction phase of morphine-induced CPP [53]. Our anatomical data demonstrate intense orexinergic innervation of NIC-activated regions of Acb; however, whether the NIC-activated cells are the targets of these orexin projections remains unclear.

## Limitations of the Current Study

In the present study, we have used c-Fos expression as a measure of neuronal activity in the mouse brain after acute administration of NIC. c-Fos has been used by us and others to assess neuronal activity after a variety of stimuli, including nicotine [6,21,22]. Thus, our data analysis is based on the assumption that NIC activation of neurons leads to induction of the c-fos gene and production of c-Fos protein. However, the possibility that a small proportion of NIC-activated cells may not have responded by induction of c-Fos and, thus, may not have been detected cannot be ruled out. Nonspecific activation of c-Fos associated with stimuli not related to NIC has been addressed by appropriate controls and habituation of animals, as described in the Methods. Nevertheless, the possibility that a small fraction of c-Fos IR cells may reflect nonspecific activation cannot be completely eliminated. Despite these possibilities, the majority of c-Fos IR cells reflect those that are activated by NIC (21) and therefore the orexinergic projections to these NIC-activated sites implies that orexinergic innervation is likely to play a role in nicotine-related responses.

## Conclusion

In summary, the present study is the first to systematically map the neuroanatomical distribution of orexinergic nerve fibers with respect to NIC-activated cells of the reward-addiction neurocircuitry. The extensive orexinergic innervation of NIC-activated brain regions such as VTA and LC supports and extends previous findings regarding the involvement of orexinergic signalling in modulating the behavioural and pharmacological effects of nicotine.
